# Middle managers’ ethos as an inner motive in developing a caring culture

**DOI:** 10.1177/09697330221140519

**Published:** 2022-12-22

**Authors:** Diako Morvati, Yvonne Hilli

**Affiliations:** Department of Health and Care Sciences, 208392 The Arctic University of Norway, Harstad, Norway; Faculty of Nursing and Health Sciences, Nord University, Bodø, Norway; Faculty of Nursing and Health Sciences, Nord University, Bodø, Norway

**Keywords:** Care ethics, leadership/management, organizational culture, nursing homes, hermeneutic, qualitative research

## Abstract

**Background:**

Middle managers play a key role in promoting a caring culture in nursing homes. However, there is limited knowledge about middle managers’ inner motives and their experiences of their responsibility in developing a caring culture.

**Research aim:**

The aim of the study is to get a deeper understanding of middle managers’ motives and their experiences of their responsibility to develop a caring culture in nursing homes.

**Research design:**

A qualitative design with a hermeneutic approach inspired by Gadamer was chosen which guided the interpretation of data. Qualitative semi-structured interviews were conducted.

**Participants and research context:**

Data were collected from thirteen middle managers in nursing homes, in six municipalities in northern Norway in September and October 2021.

**Ethical considerations:**

The study was approved by the Norwegian Centre for Research Data. Oral and written informed consent was obtained from participants.

**Findings:**

The findings show that the middle managers had non- egoistic motives to promote a caring culture as expressed in their attitudes and actions. They felt responsible to promote a caring culture where both patients and staff experienced care and were respected and recognized as unique individuals. Middle managers as good role models are responsible for being present and raising awareness of the importance of care in the nursing home culture by systematically reflecting on care values. However, a strong focus on the financial and administrative demands limits the middle managers’ possibilities to promote a caring culture and prevented them from always acting as they wanted to act, which often causes moral distress.

**Conclusion:**

Being in contact with inner motives, enables the leader to promote a homelike and caring culture where both patients and staff feels respected and recognized as unique individuals. This study highlights the importance of systematic reflection on caring values in nursing homes which leads to value awareness among all actors.

## Introduction

Nurse managers in nursing homes have a middle management position, closest to patients and personnel, often with a tripartite responsibility for personnel, finances and patient care.^1^ Their responsibilities involve caring for vulnerable patients, promoting the well-being of nurses, a good working environment, and ensuring a financially efficient healthcare organization.^2,3^ The proportion of older people in the population is increasing much faster than before. The number of people aged 80 and above in Norway may increase from 190,000 in 2000 to almost 590,000 in 2050.^4^ This will entail increasing needs for care in nursing homes and thus place greater demands on nursing home management.^5^ Norwegian primary health care has been reformed to align with new public management since the 1990s. This management ideology places efficiency and productivity demands on middle managers.^6,7^ If the financial discourse becomes the prevailing culture in nursing homes, this may be at the expense of care values.^7,8^ Nationally and internationally, there are concerns about the quality of care in nursing homes.^3,9–11^ Organizational demands, time pressure and shortages of nurses are seen as reasons for these concerns.^10,11^

Earlier studies show that middle managers play an important role in improving the quality of care^12–18^ and in promoting caring values in the organizational culture.^19–22^ A caring culture contributes to ethical growth, develops nurses’ understanding of patients’ needs and values, which can also have a positive effect on patients’ health and well-being and staff engagement, and job satisfaction.^14,15,23^ A good and appreciative leader and a good working environment were among the important factors that have encouraged Norwegian nurses to stay in the profession.^24^ According to managers, the organizational culture is important to prevent abuse against both patients and staff.^5,22,25^ Managers are responsible for promoting good attitudes, ways of thinking and ethical principles in the culture.^21,26^ Caring about the staff and caring for patients have been described by managers as two sides of the same coin.^17,18,27^ Caring has been described by nursing managers as showing trust in and respect for human beings and thus preventing abuse.^16,28^ Delegation of responsibility to registered nurses improves patient care and enhances staff engagement.^5,12,27,29^ Values such as respect for others and the establishment of good relationships are formed in the organizational culture to avoid disrespect of patients and employees.^5,22^ A culture that does not seek to understand the perspective of older people and only focuses on efficiency will lead to abuse of patients and adverse events.^25,28^ Nurse managers have a responsibility to create a caring culture where they support nurses in difficult situations. Such support will enhance nurses’ well-being in everyday clinical practice and promote a healthy work environment.^19,21,25,26^

This literature review shows that there is limited knowledge about middle managers’ inner motives and their experiences of their responsibility in developing a caring culture. Therefore, in this study, we wanted to highlight this perspective by conducting individual interviews with middle managers in nursing homes.

## Theoretical perspective

The theoretical perspective is based on the theory of caritative leadership^2^ and ethos of caring, based on Eriksson’s caritative caring, in which caring is the essence of nursing.^30–32^ The caritative caring theory is rooted in the ethos of nature. Human beings are longing for a place where they feel safe and respected by others. The basic values, the ethos, of the human being, can be understood as a moral attitude that promotes a good quality of life where the individual feels metaphorically ‘at home’ or at homeness.^32,33^ Ethos of caring comprises three dimensions that are interwoven into a pattern and are symbolically depicted as the ethos of the human being’s innermost room. When the ethos becomes visible, it is reflected in the manner of being, in the manner of conduct, and in the tone of the abstract and physical room where human beings meet and interact.^32^ The room is characterized by its culture and atmosphere, that is, how the human beings live in the metaphorical ‘home’.^32^ The ethos of caring has its basis in caritas. A caring culture is based on fundamental values such as love and mercy, emphasizing the importance of a good life, and promoting health and well-being.^30^ Caritas as a motive for leadership can awaken an inner desire to take on the responsibility to care for patients and alleviate suffering.^2^ Responsibility is viewed as part of the essence of the ethos, the basic values of human beings. The basic values form the core of a caring culture, which implies showing respect for human dignity and holiness, having the courage to take on responsibility, and being engaged.^31–33^

## Research aim

The purpose of this study is to get a deeper understanding of middle managers’ motives and their experiences of their responsibility to develop a caring culture in nursing homes.

## Research design

This study used a qualitative design with a hermeneutic approach inspired by Gadamer. According to Gadamer, human understanding is shaped by prejudice. Understanding is achieved through a dialectical process, called the hermeneutic circle or spiral. The hermeneutic circle implies a whole-part-whole approach to data analysis, involving what we are to interpret, the context this is interpreted in, and using our prejudices.^34^

### Participants

Sixteen middle managers in six municipalities of different sizes in Northern Norway were contacted. The sample was strategic, and inclusion criteria for study participation were (i) employed middle manager of a nursing home with long-term residents, (ii) at least 2 years’ experience as a manager and (iii) a minimum of a bachelor’s degree in the field of health and social sciences. One person declined and two were excluded because one had less than 2 years of experience and the other was the manager in a nursing home for short-term residents. The final sample consisted of thirteen middle managers (twelve females and one male) working in six different sized municipalities. The age range was 37–62 years, while years of management experience ranged from 3 to 22 years (Table 1).

**Table 1.** Characteristics of the thirteen participants.

Gender	Age	Management experience
Female: 12	37–62	3–22 years
Male: 1	Average: 48,3	Average: 9,7 years

### Data collection

Qualitative semi-structured in-depth interviews were chosen as the data collection method. An interview guide was used to increase awareness of prejudices and maintain the focus of the interviewer and interviewee on the topic and questions. An attempt was made to ask general, open questions, such as ‘Could you tell me about ...?’ and for deeper understanding, follow-up questions were asked, such as ‘Could you elaborate on…?’. To avoid misunderstanding questions such as ‘If I understand you correctly, it is…’ were asked. Data were collected in September and October 2021. Interviews lasted between 40 and 60 min. Due to the COVID-19 pandemic, six interviews were conducted via the Teams platform, and seven at the workplace. All interviews were conducted during working hours. Data saturation was reached after 13 interviews. No new information was received, the same themes were repeated. The interviews were audio recorded and transcribed verbatim immediately afterwards. Our data consisted of 154 A4 pages

### Data analysis

Data were analysed and interpreted using a hermeneutic method inspired by Fleming et al.^35^ The interpretation process comprises four steps: To gain an understanding of the whole, the data were read several times. In the next step, each sentence and section were examined and interpreted to discover nuances and meaning units. The sentences were related to the whole text and back again to get a deeper understanding. In the final phase, the themes that represent a shared understanding between the researcher and participants were formulated.

## Ethical considerations

This study is based on the research ethics of the Norwegian National Research Ethics Committee for Medicine and Health Sciences and the professional ethics guidelines of the Norwegian Nurses’ Association.^36,37^ The study has been approved by the Norwegian Centre for Research Data (Ref. No. 207221). Unit managers in the municipalities were contacted by e-mail and gave their permission for the study. The middle managers were informed about the study both orally and in writing. They were informed about data privacy and of the possibility to withdraw from the study at any time. Participants confirmed by signing a consent form before the interviews. All data were immediately anonymized by replacing personal information with a code (p1-p13). The audio files were deleted immediately after transcription.

## Findings

The interviews contained rich data imbued with an ethos of responsibility, engagement, respect, and recognition of patients and staff as unique human beings. The main themes were (1) responsibility as an inner motive, (2) respect for human dignity and (3) being a middle manager is like living in two different worlds (Table 2).

**Table 2.** Example of the interpretation process.

Meaning units	Sub-themes	Main themes
You must have a view of others that is humble enough, so you don’t put yourself in the center…. otherwise, you’ll keep thinking about the other in relation to yourself. (P1)	Having non- egoistic motives: Putting others first	Responsibility as an inner motive
If people say bad things about each other as colleagues or talk in a patronizing way about patients and their relatives… well then, I have to step in and say that it’s not okay. (P7)	Consistency between attitude and code of conduct by being a good role model	
I think it’s about noticing each other and if someone’s absent for example when they come back to work, you say, “Great to see you back again”… so you show you care, not just as a manager, but also as a fellow human being. (P3)	Caring about the staff	
You have to see the person, so the individuals who come and live here are allowed to be themselves with their entire life history and we treat each one as a special person. (P8)	Encouraging patients to be themselves– a unique person	Respect for human dignity
They must get something they’re responsible for, something they’re interested in that makes them say it’s important for me to go to work. (P6)	Recognizing the uniqueness of the staff	
We have systematic ethical reflection, that’s about ensuring that what we want to do is based on patient needs… we may occasionally focus on ourselves too much so that what we do is based on our own needs. (P1)	Increasing value awareness by reflection	
It’s about being available for employees, but a lot of administrative jobs go beyond the time you have available. (P3)	Strong focus on administrative demands	Being a middle manager is like living in two different worlds
…It’s like being squeezed between expectations from above and reality. (P5)	Being squeezed between economic and care values	

## Responsibility as an inner motive

Responsibility as an inner motive emerged as a pattern that included the sub-themes of having non- egoistic motives, consistency between attitude and code of conduct, and caring about the staff.

### Having non-egoistic motives: putting others first

The participants stated that to promote a caring culture, it was important to have an attitude to others that focused on the other and putting oneself aside. The managers’ responsibility was towards others, with an inner desire to promote everyday well-being based on their capabilities and needs, not the needs of the manager:

You must have a view of others that is humble enough, so you don’t put yourself in the center…. otherwise, you’ll keep thinking about the other in relation to yourself. (P1)

Responsible management in this context can mean the ability to distinguish between one’s own and others’ needs and serve the other with humility. Participants pointed out that the other could be a patient, a staff member or a relative. The managers felt responsible to promote a caring culture where patients feel they are cared for, staff have job satisfaction and relatives feel noticed:

What motivates me is that patients feel in safe hands staying here and that they’re happy daily, that the staff are happy too and that the patient’s relatives are satisfied. (P10)

The managers’ motives for being leaders seemed to be unselfish and without self-interest: ‘It’s not like if people do nothing for me, then I don’t need to do anything for them’ (P3). In other words, they were more concerned with others’ well-being than their own, which is the opposite of egoism.

### Consistency between attitude and code of conduct by being a good role model

The manager’s responsibility to set a good example was described by all participants as a vital element of promoting a caring culture: ‘I’d say that my responsibility is to lead by example’ (P7). The participants felt that leading by example meant demonstrating their attitude towards others. One way to show this attitude in action was to be with the staff and take over some of the care responsibilities: ‘If I sit here (in the office), I don’t learn much about patient needs and what the staff need help with, you learn that when you’re out doing nursing’ (P2). The managers stated that this was about demonstrating their most important values in interacting with others to appear genuine and honest:

It’s all about the kind of example I set… how I talk to my patients… how well I listen… so I have to show it. (P1)

Attitude is expressed through the manner of being and different actions. To promote a caring culture, the managers found it important to show caring values through their behaviour and their reactions to various incidents in the nursing home:

If people say bad things about each other as colleagues or talk in a patronizing way about patients and their relatives… well then, I have to step in and say that it’s not okay. (P7)

People can be hurt and offended by the way others talk to them. This manager seemed to be aware of this and noticed how people related to each other, and intervened if a situation arose that could mean someone was offended or treated with disrespect.

### Caring about the staff

The participants emphasized that staff care was closely linked to patient care. It was, therefore, crucial for the managers to care about the staff to underline the importance of care for patients:

My main task is really to take care of my staff so that they can take proper care of the residents. (P8)

There are many ways for managers to show that they care about the staff. One said: ‘I try to support them, praise them, advise them, and show that I want the best for them’ (P6). Another manager said:

I think it’s about noticing each other and if someone’s absent for example when they come back to work, you say, “Great to see you back again” … so you show you care, not just as a manager, but also as a fellow human being. (P3)

This manager seemed to have an inner desire to show that she cared about her staff and felt responsible for them as a fellow human being. The participants felt that it was important to treat the staff as unique individuals by showing consideration for their problems during periods when things were going badly for them:

If any members of staff are having problems, I try to be helpful where I can. (P11)

As a fellow human being, this manager showed care and understanding of the staff’s difficulties and acknowledged their feelings by enabling them to feel support and care.

## Respect for human dignity

Interpretation of the data reveals respect for human dignity as the core of a caring culture. It embraces the following sub-themes: Encouraging patients to be themselves, recognizing the uniqueness of the staff, and increasing value awareness by reflection.

### Encouraging patients to be themselves – a unique person

To ensure patient dignity, the participants emphasized the importance of making the nursing home like a home where patients could completely be themselves: ‘I want to make the nursing home like a home for the patients, so they feel this is home and not a hospital ward’ (P7). Home can be understood as a place where a patient feels safe and taken care of as an individual with his or her entire life history:

…You have to see the person, so the individuals who come and live here are allowed to be themselves with their entire life history and we treat each one as a special person. (P8)

Being treated as a special person can be understood as being taken care of as a unique person who is allowed to live according to his or her values ​and interests. The managers felt a responsibility to respect patients’ values ​​and accept them as they were. Important factors here were knowledge of patients’ background, what work they did and what they had been interested in, to relate to the whole person, and to help them to have a meaningful life:

We find out about the values of all new patients about their physical, mental, and spiritual needs, and then we make action plans and activities based on whatever… means something to them. (P4)

Letting patients be themselves may imply that patients are recognized and respected as the unique individuals they are, regardless of their illness and level of functioning.

### Recognizing the uniqueness of the staff

The participants explained that recognizing the uniqueness of the staff was about respecting their efforts and abilities to enable them to use their potential in their job: ‘Seeing the work each employee does here is linked to acknowledging every single one of them’ (P5). Being recognized as a unique individual meant being valued as a member of a community that worked together towards a common goal. This was expressed by giving the staff responsibility for what they were good at and interested in:

They must get something they’re responsible for, something they’re interested in that makes them say it’s important for me to go to work. (P6)

Giving the staff responsibility seemed to be crucial for them to experience belonging and meaning in their work. Further, the managers emphasized that the staff could have different abilities outside their profession that may have a great impact on patient´s well-being, and should therefore be allowed to develop the abilities they have:

They may have non-academic abilities that they’re very good at… like playing the guitar... they should use the abilities they have. (P10)

The managers seemed to value the uniqueness of the staff and recognize and respect their various skills. Enabling the staff to take responsibility and use their potential seemed to be of great importance for them to feel acknowledged.

### Increasing value awareness by reflection

Busy working days may leave less time to think about care values. The managers felt responsible for enabling open communication and reflection on care values to increase value awareness: ‘It’s about using reflection in a way to get what we do with patients right up into the part of our brains where we’re most aware of it’ (P5). Participants stated that staff could have different understandings of care values, which could constrain a caring culture. ‘What limits us is the fact that we don’t all have the same opinion about what care is’ (P2). It was thus important for managers to create space and time for regular reflection on care values to arrive at a common understanding. This could be done in different ways. Some managers would discuss challenges as they arose at work and reflect on care values at staff meetings. One manager emphasized the importance of using systematic ethical reflection:

We have systematic ethical reflection, that’s about ensuring that what we want to do is based on patient needs… we may occasionally focus on ourselves too much so that what we do is based on our own needs. (P1)

The managers felt that it was their responsibility to promote systematic reflections on caring values ​​in the nursing homes. The reflections aimed to protect the dignity of the patients and to reflect upon opinions, priorities and perspectives that dominated the culture.

## Being a middle manager is like living in two different worlds

All participants described conflicting demands and expectations from top leaders which might cause barriers to creating a caring culture in nursing homes. Being a middle manager was perceived as living in two different worlds. On the one hand, a strong focus on administrative and economic demands and on the other hand being a caring manager promotes a caring culture.

### Strong focus on administrative demands

Availability was described by all participants as an important factor in promoting a caring culture in nursing homes. However, the managers mentioned time pressure due to a strong emphasis on administrative work; this was a challenge that limited their opportunities to be available to the staff and to get to know them well:

… It is with time, that sometimes you get snowed in with emails about what should have been done, this and that… so this administrative work versus being with the staff can be difficult. (P6)

Managers seem to prefer to spend their time being present with staff and patients, but this is hindered by time pressures related to many administrative responsibilities:

…It’s about being available for employees, but a lot of administrative jobs go beyond the time you have available. (P3)

### Being squeezed between economic and care values

Participants felt that the greatest obstacle to developing a caring culture was a tight budget: ‘*The biggest challenge is our budget*’ (P13). The challenge was for managers to stay within the budget while also providing high-quality care, being a good listener, and meeting the wishes and needs of the staff:

… You have an employer who expects you to operate on a very tight budget, and then you have patients and staff who want this and that… It can be quite tough. (P7)

The managers seemed to be caught between the pressures of financial considerations and care values. Being a middle manager was therefore perceived as living in two different worlds, where expectations from leaders above did not correspond to reality:

…It’s like being squeezed between expectations from above and reality. (P5)

The managers were frustrated not getting an understanding of their difficult role. The conflicting expectations from top leaders and their engagement and inner values could lead to feelings of failure:

…You might not always get things done as well as you want… it’s a physical strain and it can be a mental strain. (P8)

Failure to live up to expectations could over time lead to feelings of inadequacy and moral distress.

## Discussion

This study aimed to get a deeper understanding of middle managers’ motives and their experiences of their responsibility to develop a caring culture in nursing homes. Middle managers highlighted how their basic values, responsibility, respect, and reverence for the uniqueness of patients and the personnel served as an inner driving force to be a committed leader in promoting a caring culture. They narrated their manner of being a leader with an inner responsibility to do good for patients and the staff.^17^ Responsible management thus involves ethos as a reflective attitude that implies an inner desire to be responsible for alleviating the suffering of others through love and mercy.^33^ This is an expression of equitable behaviour^7^ and shows that the managers were driven by non- egoistic motives where they felt a deep responsibility for the other, which stands in contrast to egoism.^2^ To promote a caring culture, the managers emphasized the importance of setting oneself aside in interaction with others. To lead by example as a way of demonstrating which attitudes and values ​were emphasized most in actions and interactions with others, which is in line with previous studies.^5,14,17,19^ The managers’ ethos became evident in their code of conduct, which enabled others to feel respected, valued and acknowledged.^32^ The managers stated that this could be expressed by giving the staff responsibility according to their level of knowledge. Giving staff responsibility can lead to commitment and development, which will help them to feel respected, belong and give meaning in the workplace, as seen in previous studies.^5,12,27,29^

As in earlier studies, this study shows that caring for patients is closely linked to caring about the staff. When managers care about their staff, they also care for their patients.^17,18,38^ Caring about one’s staff involves consideration for the staff’s professional and personal development by adopting an individualized approach to supporting them. This implies treating employees as unique individuals.^2^ Caring implies an ethical attitude that aims to protect human dignity.^32,33^ The managers emphasized the importance of making the nursing home a place where patients could feel at home and thus preserving patients’ dignity. The patients were respected and treated as unique individuals enabling them to live by their values ​​and interests. Respect for human dignity is also about recognizing the uniqueness of each member of the staff.^2,8^ This study shows that respect for human dignity is seen as a fundamental value of a caring culture. If this fundamental value changes, the whole culture will change.^33^ The ethos will set the tone in the culture, where different actors, patients and family members encounter each other. There is reciprocity, the culture is shaped in the work environment by different actors, but the prevailing culture will also shape those who work in this culture, in the metaphorical home.^32^ The managers narrated about being driven by inner values where they feel metaphorically ‘at home’. Home as ethos gives courage to an individual to be the innermost person and to follow the voice of the heart. In the innermost room, lies the driving force that gives meaning to life itself.^32^ Furthermore, the staff differed in their views and understanding of what good caring involved, which is congruent with previous studies.^25,29^ In promoting a caring culture, the managers considered it important to create time and space for continuous reflection on care values. Reflection was emphasized as a tool to become more aware of the importance of caring values. The main purpose was to protect the basic values in a caring culture, namely respect for human dignity. Like previous studies, the managers found that their main challenge in promoting a caring culture was that they felt pulled between conflicting demands and expectations.^12,13,16,29^ Being a middle manager was thus perceived as living in two different worlds with much administration and operating on a tight budget. Managers felt in conflict with their ethos and experienced failing to meet expectations from top leaders. This might lead to moral distress over time since the managers were driven by their inner basic values but had difficulties achieving the desired goals due to outer factors.^39–41^ The findings show that the basic values matter and have an impact on health, well-being, engagement and job satisfaction. It’s important that middle managers are aware of their ethos and how pivotal this is in creating a caring culture in nursing homes.^32^ Creating a caring culture should be a stated vision from top management to retain nurses and guarantee high-quality care.

## Strengths and limitations

To ensure the trustworthiness of the study, the criteria proposed by Lincoln and Guba^42^ has been used. To secure transparency, credibility and confirmability many direct quotes were used. Further, an interview guide was used to provide a clear focus during all interviews. Ongoing conversations were held between the junior and senior researchers to ensure dependability and to prevent the risk of unconscious prejudices leading to misunderstanding.^35^ Thirteen participants from six municipalities of different sizes may have strengthened the transferability of the study to other municipalities. One limitation of the study is that of 13 participants only one was male, which might have affected the findings. Male managers might have provided other nuances and perspectives.

## Conclusion

Middle manager’s ethos is about responsibility, respect, recognition, and being conscious of attitudes and actions in relation to patients and the staff. Being in contact with inner motives, a sense of at-homeness, enables the leader to promote a homelike and caring culture where both patients and staff feels respected and recognized as unique individuals. This study thus highlights the importance of systematic reflection on caring values ​​in nursing homes which leads to value awareness among all actors. An awareness of ethical values influences attitudes and affects the work environment and have positive effects on patients’ health and well-being and staff engagement, and job satisfaction. A caring culture promotes a healthy work environment for nurses to stay in the profession. The impact of a caring culture should be acknowledged by top management and politicians and taken seriously. The importance of a caring culture from the perspectives of nurses and patients should be further researched in nursing homes and other contexts.
